# Evolution of the hepcidin gene in primates

**DOI:** 10.1186/1471-2164-9-120

**Published:** 2008-03-05

**Authors:** Ludovica Segat, Alessandra Pontillo, Michele Milanese, Alessandro Tossi, Sergio Crovella

**Affiliations:** 1 Genetic Unit, IRCCS Burlo Garofolo and Department of Reproductive and Developmental Sciences, University of Trieste, Trieste, Italy; 2 Department of Biochemistry, Biophysics and Macromolecular Chemistry, University of Trieste, Trieste, Italy; 3 Department of Genetics, Federal University of Pernambuco, Recife, Brazil

## Abstract

**Background:**

Hepcidin/LEAP-1 is an iron regulatory hormone originally identified as an antimicrobial peptide. As part of a systematic analysis of the evolution of host defense peptides in primates, we have sequenced the orthologous gene from 14 species of non-human primates.

**Results:**

The sequence of the mature peptide is highly conserved amongst all the analyzed species, being identical to the human one in great apes and gibbons, with a single residue conservative variation in Old-World monkeys and with few substitutions in New-World monkeys.

**Conclusion:**

Our analysis indicates that hepcidin's role as a regulatory hormone, which involves interaction with a conserved receptor (ferroportin), may result in conservation over most of its sequence, with the exception of the stretch between residues 15 and 18, which in New-World monkeys (as well as in other mammals) shows a significant variation, possibly indicating that this structural region is involved in other functions.

## Background

Antimicrobial peptides (AMPs) are phylogenetically ancient and ubiquitous molecules that play an essential role in many aspects of host defence [1]. In plants and invertebrate animals they are central to protecting the host from infection, while in vertebrate animals that have developed an acquired immunity they not only provide a rapidly mobilised first line of defence as part of the innate immune system, but also act as signalling molecules to cellular components of both innate and adaptive immunity [2]. Being at the interface between host and commensal or pathogenic microbes, they generally undergo an accelerated evolution, which has resulted in many different AMP families and a remarkable sequence variation even within structural groups [3].

In some cases, molecules derived from AMPs have acquired functions beyond host defence, although they can maintain a role in the prevention of infection. The recently identified peptide hepcidin/LEAP-1 is a good example. It was independently isolated from human urine and plasma ultra filtrate, respectively, by two groups searching for novel AMPs. Both denominations thus reflect its hepatic expression (hep- or Liver Expressed) and its antimicrobial activity *in vitro *(-cidin, or Antimicrobial Peptide) [4,5]. Subsequently, the observation that disruption or mutation of the hepcidin gene in mouse and man results in severe forms of iron overload identified it also as a negative regulator of iron absorption, recycling and release from stores [6-8]. It functions by inducing the internalisation of the ubiquitous, vertebrate, iron export membrane protein ferroportin, at the major sites of iron flow (enterocytes, tissue macrophages and hepatocytes), causing a decrease in serum iron levels. It was thus proposed that hepcidin was the long sought iron regulatory hormone, whose synthesis is controlled by iron levels and erythroid demands [9,10]. Hepcidin maintains a role in host defence by acting as a bridge between immunity and iron-metabolism, as indicated by the fact that it is markedly induced by infection and inflammation [7,8,11]. This likely reflects an antimicrobial action via iron limitation rather than a direct antimicrobial activity [12,13].

In humans, hepcidin is encoded by three small exons in the *HAMP *gene (19q13.1), and translates to an 84aa pre-propeptide presenting a furin cleavage site. It is expressed and processed predominantly in liver for plasma delivery, and it is excreted by the kidney. In urine, the predominant form is of 25 amino acids (hep-25) with a charge of +2, but two shorter, inactive peptides (hep-22 and hep-20) were also detected [4]. It does not show significant sequence similarity to any other known AMP, but it has been associated with the defensin family of host defence peptides due to its salt-sensitive antimicrobial activity *in vitro*, cationic and amphipathic nature, and the presence of 4 disulfide bridges in its tertiary structure. However, unlike defensins, it assumes a hairpin conformation which is stabilised by four S-S bridges placed in a ladder-like manner, including an unusual and possibly reactive vicinal disulphide. Structurally, it is therefore more reminiscent of AMPs such as protegrin [8]. It is not clear whether the observed *in viv*o antimicrobial activity is biologically relevant, is simply a residue of a possible evolutionary origin as an AMP, or derives from the conserved structural characteristics imposed by its function as an iron-regulating hormone.

Hepcidin homologues and putative hepcidin precursors have been identified in several vertebrate animals, ranging from mammals and birds to amphibians and fish [11,13,14]. Homology amongst the mature peptide sequences is quite high, reflecting a high degree of structural conservation and pointing to a role as iron regulatory hormones also in non-mammalian animals. Some species however express more than one hepdicin gene, whose products may have functions other than iron regulation, and in fish they may indeed still act as AMPs [11,13]. It has in fact been hypothesised that the iron-regulatory hormones have evolved from ancient fish AMPs.

We have sequenced the hepcidin gene coding region (all three exons) in 14 non-human primate species and compared it with the human one (HSS), so as to gain information about its evolution in primates and determine whether it could usefully contribute to their phylogenetic analysis. This study was carried out in the context of a broader analysis of primate host defense peptide evolution, which has included the cathelicidin LL-37 and beta-defensins, and which has shown remarkably varied patterns of molecular evolution, ranging from conservation to positive selection for variation [15,16]. By investigating the degree of variation of the hepcidins in the closely related primate species we aimed to determine the extent of purifying selection during the evolution of this gene in primates and locate regions of variation that could provide useful information on the functional determinants of this multifunctional peptide hormone.

## Results

**Figure 1:**
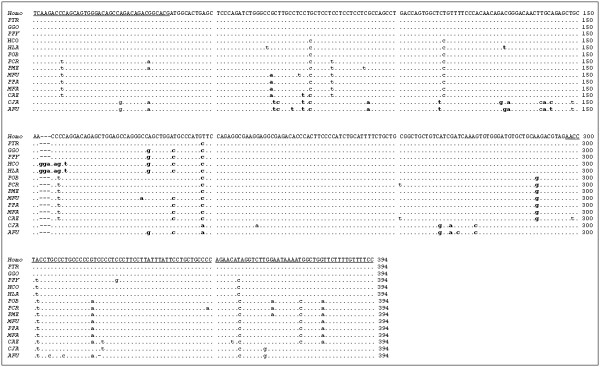
**Alignment of nucleotide sequences from coding and UTR regions of orthologous primate hepcidin genes.** The UTR regions are underlined. Dots indicate nucleotide identity with the human sequence (GeneBank accession number: NM_021175). Non-synonymous substitutions are indicated in bold.

**Figure 2:**
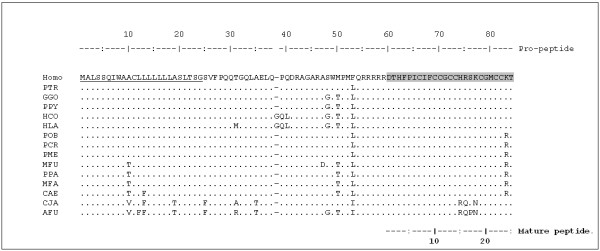
**Alignment of primate hepcidin deduced amino acid sequences.** Dots indicate residue identity with the human sequence (GeneBank accession number: NP_066998). The signal peptide region is underlined and the mature peptide sequence is highlighted in grey.

The *HAMP *gene nucleotide sequences obtained from the analyzed primate species, and the deduced corresponding amino acid sequences, are reported in Figures 1 and 2. They clearly demonstrate a high identity to the human sequence at both the nucleotide and amino acid levels. The nucleotide identity for the entire coding and untranslated (UTR) regions ranges from 99.7% with chimpanzee (PTR) to 92.6% with the NWM *Ateles fusciceps *(AFU). An in-frame insertion of three nucleotides (namely GGA, leading to the insertion of a glycine between residue 38 and 39 of the pre-propeptide) occurs in *Hylobates*.

At the amino acid level, identity ranges from 98.8% in chimpanzee (PTR) to 83.3% in NWM *Ateles *(AFU) (see Table 1).

**Table 1:** Percent identity of nucleotide (nt) and amino acid (aa) sequences calculated versus the human sequence. The highest and lowest values per each kind of identity (nt and aa) are underlined.

	**PTR**	**GGO**	**PPY**	**HCO**	**HLA**	**POB**	**PCR**	**PME**	**MFU**	**PPA**	**MFA**	**CAE**	**CJA**	**AFU**
nt identity	99.7	99.2	98.5	96.7	96.2	97.2	96.2	96.2	95.9	96.4	96.4	95.2	94.2	92.6
aa identity	98.8	96.4	96.4	92.9	91.7	97.6	97.6	97.6	94	95.2	95.2	94	88.1	83.3

**Figure 3:**
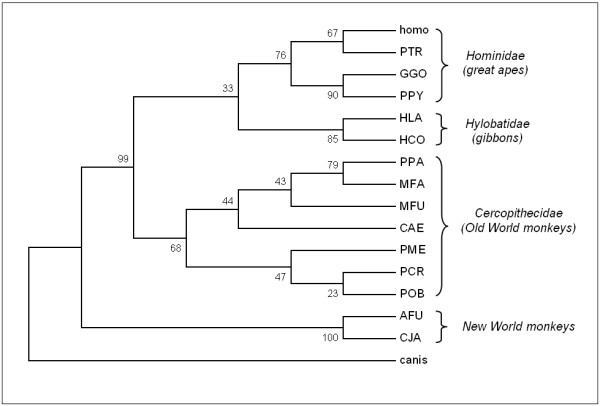
**Rooted phylogenetic tree based on primate hepcidin nucleotide sequences, prepared using the UPGMA method and using the *Canis lupus familiaris *sequence as outgroup** (GeneBank accession number NM_001007140)

The HAMP gene coding sequences were then used to compute an UPGMA tree with all primate sequences, using the *Canis lupus familiaris *HAMP gene as the outgroup [17] (Figure 3), which reflects the expected phylogenetic relationship both among families and between families. A maximum-likelihood approach, using the PAML suite of programs, was then used to determine if positive selection acted on the primate HAMP genes, and globally found that it has not (ω<1). However, a site-analysis found that individual amino acid residues may be under positive selection (ω>1), including the Gly insertion between residue 38 and 39, and Phe39Gln and Gln40Leu substitutions in the *Hylobates *sequences (see Figure 2), and the His74Arg, Arg75Gln and Ser76Pro substitution specific to the NWM sequences. A subsequent branch-analysis also indicated that although the mean ω is <1 over the entire tree, positive selection may act on the *Hylobates *and NWM branches, confirming the site-analysis data.

## Discussion

To assess whether the variations found at the amino acid level in the different primates species could have functional implications, these were analyzed in relation to the available data for the structure and activity of the human hepcidin, dividing the amino acid sequence into three parts, namely signal peptide, pro-region and mature peptide region (see Figure 2).

The entire sequence is quite conserved, but more so at the level of the mature peptide. In effect, the only significant variation in the pro-region corresponds to the insertion observed for the *Hylobates *species, whose effect is unpredictable as it falls outside the mature peptide. The putative mature peptide sequence is identical to the human one in great apes and gibbons, and shows a single conservative Lys83Arg variation in Old-World monkeys. Interestingly, a heterozygous Lys83Arg replacement leading to a peptide identical to that of the OWMs has been found in a patient with porphyria cutanea tarda and iron overload, but testing of the synthetic homologue revealed it to remain fully functional in iron regulation, so the effective role of this substitution is not yet clear [18]. Other mutations in the hepcidin gene previously reported to be associated with hemochromatosis in humans [19-21] were not found in the primate species examined (at least 5 individuals per species have been studied).

**Figure 4:**
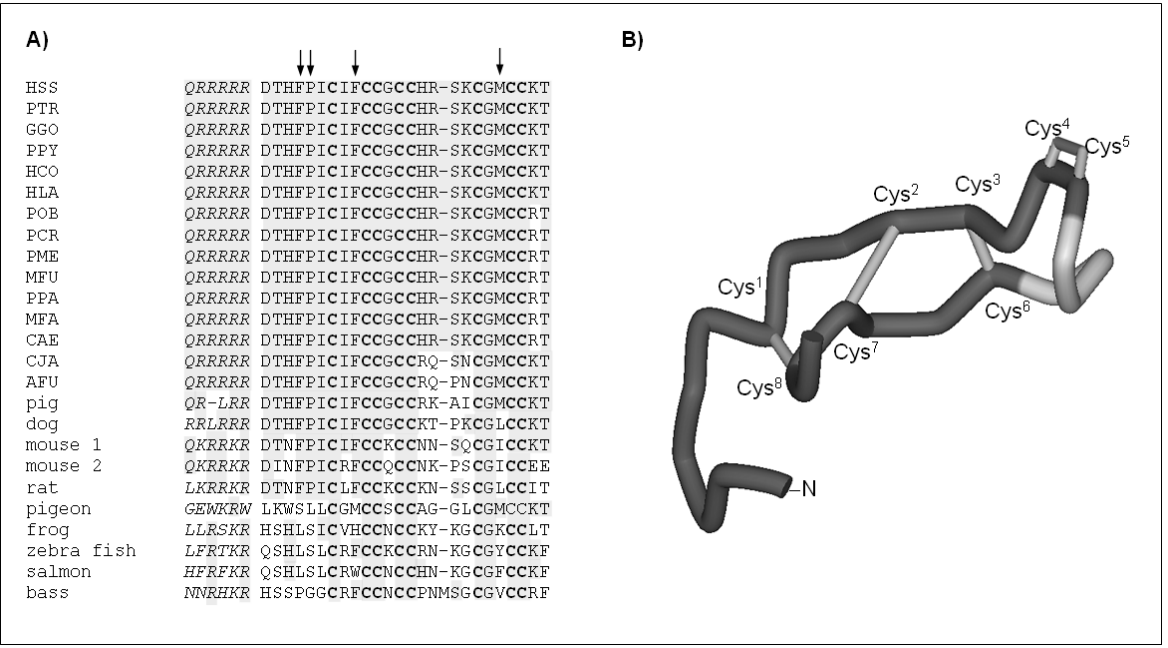
**A) Alignment of primate hepcidins with those of other mammals, amphibians and fish.** Residues identical to the human sequence are highlighted in grey. Part of the pro-peptide corresponding to the consensus propeptide convertase (furin) cleavage site is shown in italics. Arrows indicate the residues that are involved in the oligomerization of human hepcidin-25. (GeneBank accession numbers for non-primate species: *Pig *NM_214117; *Mouse-1 *BC_02158; *Mouse-2 *AY_232841; *Rat *NM_053469; *Pigeon *see ref. [14]; *Frog *DN_020182; *Zebra fish *NM_205583; *Salmon *BQ_036900; *Bass *DQ_31605). **B) **Structure of human hepcidin-25, based on the coordinate file 1M4F.pdb. The structure schematically shows the ladder-like disulphide connectivity (numbered Cys residues are indicated) and location of the variable stretch (residues 15–18) in light grey.

The pro-peptide convertase cleavage site, the functionally essential N-terminal region [18] and residues Phe4, Pro5, Phe9, and Met21 that are involved in the oligomerization of hepcidin-25 [22] are all conserved, as well as all cysteine residues that account for the formation of disulphide bond and confer hepcidin's characteristic structure. Only the New-World monkeys show significant variation in a stretch between residues 15 and 18 of the mature peptide sequence (74His, 75Arg and 76Ser, using the full pro-peptide sequence numbering) (see figure 3) which may be under positive selection. Alignment of the primate sequences with those of other mammals, as well as bird, amphibian and fish sequences [11,13,14], indicate that this region is in fact the most variable also with respect to other mammalian sequences (see figure 4a). This stretch is just C-terminal to the two Cys residues forming the vicinal disulphide at the peptide's hairpin turn (see figure 4b) [22], which may be functionally significant. While the conserved N-terminal region is recognised to be essential for interaction with ferroportin [18], the role of the C-terminal side is not well defined.

Shi et al [13] have hypothesized that, during vertebrate evolution, hepcidin differentiated its functions towards an iron-regulatory hormone starting from a more ancient peptide characterized by antimicrobial activity. In this respect, it is interesting to note that substitutions in the variable region, concerning both primates and other animals, often concerns charged residues (see Figure 4); moreover, it has been shown for different types of AMPs that variation in charge are also favoured by positive selection [15,23]. Whereas for the latter peptides, charge modulation can be directly related to the efficiency of interaction with microbes, thus affecting antimicrobial activity, in the case of hepcidin, the effect of this variation on function/s is an open question.

## Conclusion

Our results indicate that hepcidin is quite conserved in all primate species. This may derive from the fact that its role as an iron regulating hormone requires interaction with the conserved vertebrate iron transporter ferroportin [24]. In this context, while some types of residue variations, such as those observed in mouse hepcidin 2 [13], can result in a loss of the iron regulatory role, other variations, such as those observed in the stretch between residues 15 and 18 in NWM and other mammals, can more freely occur without apparently disrupting the hormonal function. It remains to be established whether these residues are involved in other possible functions of the hepcidin peptides, consistent with the possibility that they evolved under positive selection.

## Methods

### Samples

We have analyzed the *HAMP *gene in three species of great apes [*Pan troglodytes *(PTR), *Gorilla gorilla *(GGO), *Pongo pygmaeus *(PPY)], two species of Hylobatidae [*Hylobates lar *(HLA) and *Hylobates concolor *(HCO), now classified as *Nomascus concolor*], seven species of Cercopithecidae [Old World Monkeys (OWM) ; *Presbytis obscurus *(POB), *Presbytis cristata *(PCR), *Presbytis melalophos *(PME), *Papio papio *(PPA), *Macaca fascicularis *(MFA), *Macaca fuscata *(MFU), *Cercopithecus aethiops *(CAE)], and two Platyrrhine species [New World monkeys (NWM); *Callithrix jacchus *(CJA), *Ateles fusciceps *(AFU)].

We tried to minimize this risk of nucleotide intra-specific variations, by analyzing more than one individual for each species and determining a consensus sequence. This method allowed us to clear our data for single nucleotide polymorphisms variations in most species.

### DNA extraction

Genomic DNA was extracted from hairs, liver, and muscle tissues of primates as described previously by Del Pero et al. [25] and Boniotto et al. [26], using the DNeasy Tissue kit (Qiagen).

### PCR and sequencing

Genomic DNA amplification of the hepcidin three exons was performed using primers (see Table 2), designed on the basis of the human sequence available in GenBank (NM_021175) using the Primer Express 2.0 software (Applied Biosystems, Foster City, CA). The PCR reactions were carried out in a Thermal cycler 9700 (Applied Biosystems) using PCR Buffer 1×, 1 unit of Taq Gold, 0.4 mM dNTPs and variant concentration of MgCl_2 _(from 1.5 to 3 mM). The amplification conditions were 30 sec at 95°C, 30 sec at variable annealing temperature between the different templates (from 52°C up to 60°C), 30 sec at 72°C for 35 cycles. PCR products were observed, under UV light, in a 2% agarose gel, stained with ethidium bromide. DNA sequencing of PCR products was performed using the BigDye Terminator Cycle Sequencing Ready Reaction Kit v. 2.0 (Applied Biosystems). DNA sequences were detected and analyzed on an automated ABI Prism 3100 Genetic Analyser (Applied Biosystems).

**Table 2:** Primers used to perform the genomic DNA amplification. Additional couples of primers (e.g. 1N, 1NN and 2-3N) were used for each amplicon as it was not possible to amplify DNA from all the primates using a single set (e.g. 1 and 2–3).

***EXON***	***FORWARD PRIMER***	***REVERSE PRIMER***
**1**	5' tctctcccgccttttcgg 3'	5' tgaggcctggctctccc 3'
**1N**	5' gccttttcggcgccac 3'	5' agacgtcctgagctctgctca 3'
**1NN**	5' ccccataaaagcgactgtcac 3'	5' ctcccatccctgctgcc 3'
**2–3**	5' gtttaaaccacttggagaggagca 3'	5' acactcggcagagagaaaggac 3'
**2-3N**	5' gaggtccactgggcccc 3'	5' acatgacccaccaagcactg 3'

### Sequence alignment and Data Analysis

Multiple alignment of the nucleotide and amino acid sequences were performed using the program CLUSTAL W [27].

A phylogenetic tree was obtained by the UPGMA method, using the PHYLIP software package [28]. Maximum Likelihood analysis was performed with the PAML software package [29] by comparing the log-likelihood ratios of the data using different NS-sites models [30]. This type of analysis is able to identify amino acid residues with high posterior probabilities (greater than 0.95) of having evolved under positive selection. ω ratios for the tree were calculated using different models (free ratio, two- and three ratio) [31].

#### GeneBank accession numbers for non-human primate species

*Pan troglodytes *(PTR): EU076436; *Gorilla gorilla *(GGO): EU076444; *Pongo pygmaeus *(PPY): EU076437; *Hylobates concolor *(HCO): EU076448; *Hylobates lar *(HLA): EU076449; *Presbytis obscurus *(POB): EU076439; *Presbytis cristata *(PCR): EU076441; *Presbytis melalophos *(PME): EU076440; *Papio papio *(PPA): EU076438; *Macaca fascicularis *(MFA): EU076443; *Macaca fuscata *(MFU): EU076442; *Cercopithecus aethiops *(CAE): EU076446; *Callithrix jacchus *(CJA): EU076445; *Ateles fusciceps *(AFU): EU076447.

## Authors' contributions

LS performed the molecular genetic studies and participated in data interpretation; MM carried out the molecular genetic studies; AP performed the sequence alignment and evolutionary analysis; AT participated in the study design and sequence analyses and wrote the manuscript. SC conceived the study, and participated in its design and coordination and revised the manuscript. All authors read and approved the final manuscript.
